# Adequate iodine nutrition and higher salt intake in Chinese adults aged 18–59 years recommended by international organizations

**DOI:** 10.1038/s41598-024-57892-4

**Published:** 2024-03-24

**Authors:** Diqun Chen, Ying Ye, Ying Lan, Meng He, Jiani Wu, Lijin Wang, Zhihui Chen

**Affiliations:** 1 Institute for Endemic and Chronic Non-Communicable Diseases, Fujian Center for Disease Control and Prevention, Fuzhou, 350012 Fujian China; 2 School of Public Health, Fujian Medical University, Fuzhou, 350122 Fujian China

**Keywords:** Daily iodine intake, Daily salt intake, Urinary iodine concentration, Urinary sodium concentration, Nutrition disorders, Ecological epidemiology, Risk factors

## Abstract

Iodine deficiency and excessive salt intake have adverse health effects. This study evaluated the iodine level and salt intake in Chinese adults aged 18–59 years after implementing the salt reduction program and compared with both the World Health Organization (WHO) and Chinese recommendations. Adults aged 18–59 years were randomly selected using multi-stage stratified random sampling in coastal urban area (CUA), non-coastal urban area (Non-CUA), coastal rural area (CRA), and non-coastal rural area (Non-CRA) of Fujian Province, China. Iodine, sodium, and creatinine concentrations in spot urine samples were measured. Knudsen equation was used to determine 24-h urinary iodine and sodium excretion. The median urinary iodine concentration (mUIC) and urinary sodium concentration (mUNaC) among adults (*n* = 3513) were 132.0 μg/L and 4.0 g/d, respectively. The mUIC and median daily iodine intake in CUA, Non-CUA, CRA and Non-CRA were 112.1, 127.5, 128.5, 167.5 μg/L and 189.6, 182.5, 199.4, 236.0 μg/d, respectively. The mUNaC and median daily salt intake (mDSI) in these four areas were 2.4, 2.8, 2.9, 2.9 g/L and 9.8, 10.4, 10.4, 10.6 g/d, respectively. The mUIC and DII of residents were higher in the Non-CRA than in the other three areas (*P* < 0.05). The UNaC and DSI of residents were lower in the CUA than in the other three areas (*P* < 0.05). The logistic regression demonstrated that the people living in CUA and Non-CUA consumed less salt compared with those in Non-CRA. Except for Non-CUA, the DII was lower (< 150 μg/d) among women of childbearing age in the low–salt intake group (< 5 g/d) compared with the high–salt intake group (≥ 5 g/d) (*P* < 0.05). Iodine nutrition in Chinese adults aged 18–59 years was sufficient, but the salt intake was substantially higher than the WHO and Chinese recommendations. Further policy implementation is needed to reduce salt intake and improve the monitoring of iodine levels in Chinese adults, especially in women of childbearing age.

## Introduction

A lack of iodine can lead to a spectrum of growth, developmental, and functional morbidities across the entire life cycle, which are known as iodine deficiency disorders (IDDs)^1^. The World Health Organization (WHO) has recommended that household salt be fortified with iodine as a safe and effective strategy for preventing and controlling IDD, and also suggested the content for fortifying food-grade salt with iodine according to the estimated salt consumption^2^. In 2021, 124 countries legislated mandatory salt iodization, and at least 21 countries had legislation allowing voluntary salt iodization^3^. The number of iodine-deficient countries decreased from 54 in 2003 to 21 in 2021.

In the 1970s, IDD was a severe public health problem in China, with 35 million patients with endemic goiter (thyroid enlargement) and 250,000 patients with typical endemic cretinism (dullness, pygmyism, deafness, dumbness, and paralysis)^4^. In 1990, universal salt iodization was proposed to be the best way for IDD prevention and control^5^, and in 1995, it was implemented throughout China. In 2011, the average iodine content in salt was stipulated to be 25 mg/kg, and the allowable variation range of iodine content in salt was ± 30% of the average level in Fujian Province, China^6^. Along with this adjustment, the iodine nutrition level in Chinese nonpregnant population in Fujian Province was adequate (children: 100–299 µg/L, adults:1 00–199 µg/L, lactating wome: ≥ 100 µg/L) in 2017. In 2017, the median urinary iodine concentration (mUIC) was 218.2 µg/L in school-age children, 166.1 µg/L in adults, 141.5 µg/L in pregnant women, and 113.0 µg/L in lactating women^7^. Recent studies have found a better thyroid profile among pregnant women who consumed sufficient iodine regularly before becoming pregnant than those who began iodine supplementation after becoming pregnant^8,9^. Hence, it is equally important that women of childbearing age have adequate iodine intake, especially those planning pregnancy.

Many studies reported that long-term, excessively high salt consumption increased blood pressure, which is a major risk factor for noncommunicable chronic diseases, including hypertension, cardiovascular disease, stroke, and chronic kidney disease^10^. In 2013, the WHO published public health strategies for salt reduction and iodine fortification with the aim of a 30% reduction in the population’s salt intake^11^. The data collected during the China Health and Nutrition Survey (CHNS) showed that the median sodium intake among adults aged 18 years and above in China as a whole decreased from 6.4 g/d in 1991 to 5.6 g/d in 2012 and 5.3 g/d in 2018^12^. However, the amount still exceeded the WHO recommendations (< 2 g/d). In China, 63.3% of dietary sodium was from salt alone added in home cooking, and sodium intake was also among the highest globally^13^. A recent study showed that the median sodium intake in Chinese adults in Fujian was 6.9 g/d from 2009 to 2011, equivalent to 17.6 g/d of total salt, which far exceeded the WHO recommendations (< 5 g/d)^14^. High sodium intake persists due to addition of salt and other seasonings during food preparation,and increasing consumption of processed food^14^. The Healthy China Promotion Committee issued the Healthy China Action Plan (2019–2030) at the national level, establishing a target salt intake of less than 5 g/d by 2030^15^. Moreover, an action group called Action on Salt China has been set up to develop and implement an evidence-based, comprehensive, effective, and sustainable national salt reduction program to help achieve a long-term target of less than 5 g/d^16^.

As explained earlier, previous iodine deficiency surveys in Fujian showed that the iodine level was optimal among adults but the salt intake was extremely high. However, these surveys were old with small samples, and recently, policy actions to reduce salt intake had been implemented^16^. The lifestyles and dietary habits have changed, and health consciousness has increased among Chinese residents^17^. Further, there are concerns that salt reduction will induce iodine intake levels below the amount required by the human body, which in turn leads to iodine deficiency. It is crucial in public health programs as well as monitoring to consider both salt intake and iodine level of the population, as policy efforts to reduce salt intake may influence iodine level and vice versa. We launched an ongoing cross-sectional study in 2021, which was conducted in a 2-year cycle. The study aimed to evaluate the iodine level and salt intake in Chinese adults aged 18–59 years, especially women of childbearing age, after implementing the salt reduction program. The study also aimed to compare the results with the WHO and Chinese recommendations.

## Materials and methods

### Study design and participants

The target population comprised healthy adults aged 18–59 years who had lived in Fujian Province for at least 6 months during the last year. The exclusion criteria were those who were pregnant, lactating, used iodinated contrast agents within three months, had a history of radiation exposure, had a positive history of thyroid abnormalities, had severe infectious disorders, chronic diseases, and renal or other systemic diseases and had difficulties to fully communicate.

This study was launched in 2021 and is ongoing (2-year cycle), with cross-sectional surveys that use a multi-stage, stratified random sampling method to obtain a representative sample of the Fujian population aged 18–59 years. The formula for sample size in stratified random sampling, *n* = *z*^2^ × *S*^2^ × *def*/*d*^2^, was used to calculate the sample size required for analysis, including sample size (n), confidence interval (*z*), standard deviation (*S*), design efficiency (*def*) and allowable error (*d*)*.* We defined the two-sided significance levels as *α* = 0.05, power (1 − β) = 0.8, and *z*_α/2_ = 1.96. The *def* value of the stratified random sampling was 2. We needed at least 2880 participants based on the variation in sodium intake. Considering 20% would be lost to follow-up and refuse to visit, we recruited 3600 participants aged 18–59 years into the study. A total of 3600 respondents were selected for a spot urine sample collection. Ultimately, 87 samples were excluded due to incomplete collections (42 no urinary samples and 45 insufficient urinary samples), with 3513 samples remaining for the final analysis.

The Fujian Province was categorized into coastal urban area (CUA), non-coastal urban area (Non-CUA), coastal rural area (CRA), and non-coastal rural area (Non-CRA) based on the nonagricultural population, migrant agricultural population, and geographical location^7^. The number of sampling districts of each layer for the CUA, Non-CUA, CRA, and Non-CRA was 6, 5, 4, and 3, respectively, based on the total population of each layer. In each selected district, five townships were randomly selected from five different geographical locations (east, west, south, north, and center). Further, 40 residents were randomly selected from 4 age groups (18–29, 30–39, 40–49, and 50–59 years), with each group comprising 5 men and 5 women.

The study was conducted according to the guidelines of the Declaration of Helsinki and approved by the Ethics Committee of Fujian Province Center for Disease Control and Prevention (No.2020032). All participants were informed of the study before enrolling and signed a written consent form.

### Questionnaires

The basic information, assessment of the inclusion criteria, frequency and intake of iodised salt, foods (refers to iodine content more than 10 mg/100 g, and dietary iodine contribution of more than 1%), and preparations were collected through questionnaires^18–20^.

### Physical examination

The physical examination was conducted directly by trained health workers using a standard protocol. It consisted of the measurement of height and weight. The body weight was measured to the nearest 0.1 kg. The body height was measured to the nearest 0.1 cm following the Frankfort horizontal plane for the head position. Before use, all physical examination equipment underwent certification and qualification to ensure their accuracy and reliability.

### Sample collection

A 50-g household cooking salt sample and a 50-mL urine sample were collected from each participant in the afternoon. All urine samples were sub-packed and stored frozen at − 20°C, transported to Fujian Province Center for Disease Control and Prevention by cold chain vehicle and analyzed within one month of collection.

### Laboratory analyses

The iodine content in the salt samples was determined using the general test method of the salt industry^21^.The iodine concentrations in the urine samples were determined using an arsenic-cerium catalytic spectrophotometer (WS/T107-2016)^22^. Iodine measurements of all samples were performed at Fujian Province Center for Disease Control and Prevention and met the quality control requirements of the National Reference Laboratory for Iodine Deficiency Disorders. We use nationally certified reference Standard Substances for quality control. Only when all measured values of the reference substance are controlled can the test results be accepted. The standard curve’s correlation coefficient had to be greater than 0.999.

The urinary sodium concentration (UNaC) and creatinine concentration (UCC) were analyzed on the cobas 8000 analyzer (Roche Diagnostics GmbH, Mannhein, Germany). The creatinine concentrations were analyzed using an enzymatic method. The sodium concentrations were determined using ion-selective electrodes (ISE Indirect Gen.2; Roche Diagnostics GmbH).

### Definition and classification of relevant indicators

Salt was categorized into three types according to the household cooking salt iodine concentration (SIC) standard in Fujian: (1) noniodized salt with an iodine content < 5 mg/kg; (2) low-iodine salt, 5 mg/kg ≤ iodine content < 18.0 mg/kg; and (3) qualified iodized salt with an iodine content ≥ 18.0 mg/kg. The iodized salt usage rate was the amount of salt consumed with an iodine content ≥ 5.0 mg/kg in all samples. The qualified iodized salt usage rate was the amount of salt consumed with an iodine content ≥ 18.0 mg/kg in all samples.

The body mass index (BMI) was categorized into four levels based on the criteria recommended by the National Health and Family Planning Commission of the People’s Republic of China. These were underweight (BMI: < 18.5 kg/m^2^), normal (BMI: 18.5–23.9 kg/m^2^), overweight (BMI: 24.0–27.9 kg/m^2^), and obese (BMI: ≥ 28.0 kg/m^2^).

The iodine nutritional status of adults was determined based on the WHO recommendations^1^. Insufficient iodine intake was defined as mUIC < 100 μg/L; adequate iodine intake as mUIC 100–199 μg/L; iodine intake above the requirement as mUIC 200–299 μg/L; and excessive iodine intake as mUIC ≥ 300 μg/L. The age limit of women of childbearing age was considered 18–49 years based on the WHO recommendations^1^.

### Iodine and salt intake calculations

The daily iodine intake (DII) and salt intake (SDI) in adults aged 18–59 years were calculated using the following formulas:

1 $$ {\text{Daily}}\;{\text{iodine}}\;{\text{intake}}\;({\text{DII}})\;({\mu g}/{\text{d}}) = \frac{{{\text{UIC}}({\mu g}/{\text{L}})}}{{{\text{UCC}}({\text{g}}/{\text{L}})}} \times {\text{UCC}}\;{\text{reference}}\;{\text{values }}\left( {{\text{g}}/{\text{d}}} \right) \div 0.{92} $$

2 $$ {\text{Daily}}\;{\text{sodium}}\;{\text{intake}}\;({\text{DNaI}})\;({\text{g}}/{\text{d}}) = \frac{{{\text{UNaC}}({\text{g}}/{\text{L}})}}{{{\text{UCC}}({\text{g}}/{\text{L}})}} \times {\text{UCC}}\;{\text{reference}}\;{\text{values}}\;\left( {{\text{g}}/{\text{d }}} \right) \div 0.{92} $$

3 $$ {\text{Daily}}\;{\text{salt}}\;{\text{intake}}\;({\text{DSI}})\;({\text{g}}/{\text{d}})\frac{{{\text{DNaI}}({\text{g}}/{\text{d}})}}{23.0} \times {58}.{5} $$

where “0.92” represents iodine absorption^23,24^. The Knudsen equation was used to estimate 24-h urinary iodine excretion (UIE) among Chinese adults, which was the same as in Andersson ’s study in Switzerland^25^. Furthermore, our previous study showed that the Knudsen equation was the best to estimate the 24-h UIE among Chinese adults except in the morning^26^. The 24-h sodium excretion was calculated like iodine^25,27^^.^

### Statistical analysis

Data processing and statistical analyses were conducted using Excel (2019 edition, Microsoft) and IBM SPSS version 25 software (IBM Corp., Armonk, NY, USA). Normally distributed data were expressed as the means plus standard deviations, and nonparametric data were expressed as the median and interquartile range (IQR, Q_1_–Q_3_). We considered the following confounders and adjusted for these: Weights of sampling design, age, gender stratification, and lack of answers. Gender was categorized into two groups: males and females. Age was categorized into 18–49 years and 50–59 years.The UIC and UNaC among different population groups were compared using the Wilcoxon rank-sum test or the Kruskal–Wallis test. The median salt intakes in different population groups were compared using the *t* tests or analysis of variance. Linear regression analysis was conducted to compare the salt intakes in different areas, adjusting for age, gender, and BMI. The multivariate logistic regression (forward stepwise) was used to assess factors associated with high salt intake. The criterion for inclusion in the regression model was 0.05, and the criterion for exclusion was 0.1. A *P* value < 0.05 indicated a statistically significant difference. The odds ratio (OR) and 95% confidence interval (CI) were calculated. The data on salt intake were used in both univariate and multivariate linear regression models.

### Ethical approval and consent to participate

The study was conducted according to the guidelines of the Declaration of Helsinki and approved by the Ethics Committee of Fujian Provincial CDC (No.2020032). All participants were informed of the study before enrolling and signed a written consent form.

## Results

### Characteristics of the study participants

The characteristics of the 3513 respondents 1750 (49.8%)from men and 1763 (50.2%) from women) are listed in Table 1. The mean age and BMI of the adults were 41.3 ± 10.7 years and 24.7 ± 5.3 kg/m^2^. The median salt iodine concentration was 24.1 (22.6, 25.4) mg/kg.

**Table 1:** Characteristics of the study participants.

Variables	Pooled
Age (years)	41.3 ± 10.7
BMI (kg/m^2^)	24.7 ± 5.3
Salt iodine concentration (SIC, mg/kg)	24.1 (22.6, 25.4)
Coverage rate of iodized sal (CRIS, n (%))	3144 (89.5)
Consumption rate of qualified iodized salt (CRQIS, n (%))	3079 (87.6)
Urinary creatinine concentration (UCC, g/L)	1.1 (0.6,1.6)
Urinary iodine concentration (UIC, µg/L)	132.0 (77.8, 203.3)
Daily iodine intake (DII, µg/d)	195.7 (132.6, 295.8)
Urinary sodium concentration (UNaC, g/L)	2.7 (1.7, 3.8)
Daily sodium intake (DNaI, g/d)	4.0 (2.6, 5.9)
Daily salt intake( DSI, g/d)	10.2 (6.7, 14.9)

The CRIS and CRQIS were the ratios of iodized salt or qualified iodized salt to the total number of salt samples tested.

### Distribution of daily iodine intake

The mUIC and mDII among adults were 132.0 μg/L and 195.7 μg/d, respectively. There were 9.2% adults of DII below EAR. No significant differences were observed in the DII between the age and sex groups (*P* > 0.05) (Table 2). Further, the differences were not significant in women of childbearing age (188.6 µg/d) compared with women aged 50–59 years (193.2 µg/d) (*P* > 0.05). The highest mDII (236.0 µg/d) was found in the non-coastal rural area compared with the other three areas (189.6, 182.5, 199.4 µg/d) (*P* < 0.05). The lowest mDII (179.7 µg/d) was found in the underweight group compared with the other three BMI groups (197.0, 197.3, 184.1 µg/d) (*P* < 0.05). The lower mDII (166.3 µg/d) was found in the noniodized salt group compared with the adequately iodized group (208.1, 199.0, 187.9 µg/d) (*P* < 0.05).

**Table 2:** Distribution of DII based on sociodemographic and anthropometric data.

Variables	N (%)	Urinary iodine concentration(UIC) Median(IQR) (ug/L)	Daily iodine intake (DII)	
			Median (IQR) (ug/d)	< EAR (%)
Pooled	3513 (100.0)	132.0 (77.8, 203.3)	195.7 (132.6, 295.8)	323 (9.2)
Gender				
Male	1763 (50.2)	134.8 (79.0, 207.9)	202.1 (134.5, 305.7)	155 (8.9)
Female	1750 (49.8)	129.5 (76.4, 196.9)	190.9 (129.4, 285.0)	168 (9.5)
Female (18–49 years)	1325 (61.4)	133.7 (80.4, 203.6)	188.6 (129.8, 282.0)	90 (8.3)
Female (50–59 years)	681 (38.6 )	135.7 (75.0, 215.9)	193.2 (124.6, 287.0)	78 (11.5)
Age (years)				
18–49	2046 (58.2)	134.5 (80.4, 201.9)	192.3 (133.3, 288.5)	175 (8.6)
50–59	1467 (41.8)	128.1 (73.8, 204.1)	199.2 (132.0, 304.7)	148 (10.1)
BMI				
Underweight (< 18.5 kg/m^2^)	194 (5.5)	118.1 (82.0, 212.4)	179.7 (121.8, 285.0)^a^	23 (11.9)
Normal (18.5–23.9 kg/m^2^)	1957 (55.7)	130.1 (74.8, 200.2)	197.0 (133.8, 299.1)	175 (8.9)
Overweight (24.0–27.9 kg/m^2^)	1078 (30.7)	137.2 (82.2, 207.9)	197.3 (135.4, 300.9)	86 (8.0)
Obese (≥ 28.0 kg/m^2^)	283 (8.1)	134.2 (74.7, 203.2)	184.1 (114.0, 270.1)	39 (13.8)
Region				
Coastal urban area	1185 (33.7)	122.1 (73.3, 187.4)	189.6 (134.4, 273.8)	80 (6.8)
Non-coastal urban area	1002 (28.5)	127.5 (75.5, 189.5)	182.5 (122.0, 289.4)	128 (12.8)
Coastal rural area	746 (21.2)	128.5 (72.1, 203.3)	199.4 (127.2, 293.5)	93 (12.5)
Non-coastal rural area	580 (16.5)	167.5 (101.2,241.1)^a^	236.0 (159.7, 371.2)^a^	22 (3.8)
Salt iodine content				
Non-iodized	369 (10.5)	104.4 (63.3, 180.8)^a^	166.3 (112.0, 274.8)^a^	53 (14.4)
Inadequately iodized	47 (1.3)	128.6 (72.2, 159.4)	208.1 (136.8, 258.1)	1 (2.1)
Adequately iodized	3079 (87.6)	134.6 (79.7, 206.7)	199.0 (135.1, 300.7)	267 (8.7)
Excessively iodized	18 (0.5)	144.6 (69.6, 244.8)	187.9 (136.7, 386.0)	2 (11.1)

^a^Statistically significant difference in participants in the other three groups by the Kruskal–Wallis test (*P* < 0.01).

*DII* daily iodine intake, *Female (18–49 years)* women of childbearing age, *IQR* interquartile range, *UIC* urinary iodine concentration.

### Distribution of daily salt intake

The mUNaC, mDNaI and mDSI among adults were 2.7 g/L, 4.0 g/d and 10.2 g/d, respectively. No significant differences were observed in DSI among different sex, BMI, and salt iodine content groups (*P* > 0.05) (Table 3). The mDSI was lower (10.1 g/d) in adults aged 18–49 years compared with adults aged 49–59 years (10.4 g/d) (*P* < 0.05). However, the differences were not significant in women of childbearing age (9.9 g/d) compared with women aged 50–59 years (9.8 g/d) (P > 0.05). The highest mDSI (10.6 g/d) was found in the non-coastal rural areas compared with the other three areas (9.8, 10.4, 10.4 g/d) (*P* < 0.05).

**Table 3:** Distribution of daily sodium and salt intake based on sociodemographic and anthropometric data.

Variables	N (%)	Urinary sodium concentration (UNaC) Median (IQR) (g/L)	Daily sodium intake (DNaI) Median (IQR) (g/d)	Daily salt intake(DSI)	
				Median (IQR) (g/d)	N (% ) < 5g/d
Pooled	3513 (100.0)	2.7 (1.7, 3.8)	4.0 (2.6, 5.9)	10.2 (6.7, 14.9)	458 (13.6)
Gender					
Male	1750 (47.8)	2.7 (1.7, 3.7)	4.2 (2.7, 6.1)	10.6 (6.9, 15.5)	241 (13.7)
Female	1763 (50.2)	2.7 (1.7, 3.9)	3.9 (2.5, 5.6)	9.9 (6.5, 14.4)	217 (12.4)
Female (18–49 years)	1325 (61.4)	2.7 (1.7,3.9)	3.9 (2.6, 5.7)	9.9 (6.5, 14.4)	180 (13.6)
Female (50–59 years)	681 (38.6 )	2.7 (1.6, 4.0)	3.8 (2.6, 5.6)	9.8 (6.5, 14.2)	93 (13.7)
Age (years)					
18–49	2046 (58.2)	2.6 (1.6, 3.7)^a^	4.0 (2.6, 5.7)^a^	10.1 (6.6, 14,6)^a^	276 (13.5)
50–59	1467 (41.8)	2.8 (1.7, 3.9)	4.1 (2.7, 6.0)	10.4 (6.8, 15.2)	182 (12.4)
BMI					
Underweight (< 18.5 kg/m^2^)	194 (5.5)	2.7 (1.6, 3.7)	3.7 (2.5, 5.6)	9.5 (6.4, 14.4)	32 (16.5)
Normal (18.5–23.9 kg/m^2^)	1957 (55.7)	2.7 (1.6, 3.8)	4.0 (2.6, 5.8)	10.1 (6.6, 14,8)	260 (13.3)
Overweight (24.0–27.9 kg/m^2^)	1078 (30.7)	2.9 (1.8, 3.9)	4.2 (2.7, 6.0)	10.6 (6.8, 15.2)	134 (12.4)
Obese (≥ 28.0 kg/m^2^)	283 (8.1)	2.9 (1.8, 4.1)	4.1 (2.8, 5.7)	10.4 (7.2, 14.5)	32 (11.3)
Region					
Coastal urban area	1185 (33.7)	2.4 (1.5, 3.5)^b^	3.8 (2.6, 5.6)^b^	9.8 (6.6, 14.2)^b^	168 (14.2)
Non-coastal urban area	1002 (28.5)	2.8 (1.8, 3.8)	4.1 (2.6, 6.1)	10.4 (6.6, 15.4)	143 (14.3)
Coastal rural area	746 (21.2)	2.9 (1.8, 4.0)	4.1 (2.6, 5.9)	10.4 (6.6, 15.0)	92 (12.3)
Non-coastal rural area	580 (16.5)	2.9 (1.8, 3.9)	4.2 (2.9, 6.1)	10.6 (7.3, 15.4)	55 (9.5)
Salt iodine content					
Non-iodized	369 (10.5)	2.6 (1.7, 3.7)	4.1 (2.7, 6.1)	10.4 (6.7, 15.4)	52 (14.1)
Inadequately iodized	47 (1.3)	2.5 (1.5, 3.6)	4.2 (2.6, 6.2)	10.6 (6.5, 15.7)	3 (6.4)
Adequately iodized	3079 (87.6)	2.7 (1.7, 3.8)	4.0 (2.6, 5.8)	10.2 (6.7, 14.8)	401 (13.0)
Excessively iodized	18 (0.5)	3.2 (1.7, 3.9)	4.2 (3.3, 5.5 )	10.8 (8.5, 14.1)	2 (11.1)

^a^Statistically significant difference in participants aged 50–59 years by the Wilcoxon rank-sum test (*P* < 0.01).

^b^Statistically significant difference in participants who lived in the other three areas by the Kruskal–Wallis test(*P* < 0.01).

*Female (18–49 years)* women of childbearing age, *IQR* interquartile range, *UNaC* urinary sodium concentration, *DNaI* daily sodium intake, *DSI* daily salt intake.

### Factors associated with high salt intake

Based on the DSI, the participants were categorized into two groups: the low–salt intake group (< 5 g/d) and the high–salt intake group (≥ 5 g/d). Multivariate logistic regression analyses were conducted using sex, age, region, and BMI as independent variables and DSI as the dependent variable (Table 4). Compared with people living in a non-coastal rural area, those living in coastal and non-coastal urban areas consumed less salt (OR = 0.639, 95% CI 0.462–0.884, *P* = 0.007; OR = 0.634, 95% CI 0.456–0.883, *P* = 0.007). No significant differences were observed in the other independent variables.

**Table 4:** Factors associated with high salt intake.

Variables	N	Β	OR	95% CI	*P*
Gender					
Female	1763	Reference			
Male	1750	0.087	1.091	0.893–1.334	0.394
Region					
Non-coastal rural area	580	Reference			
Coastal urban area	1185	− 0.448	0.639	0.462–0.884	0.007
Coastal rural area	1002	− 0.455	0.634	0.456–0.883	0.007
Non-coastal urban area	746	− 0.286	0.751	0.527–1.071	0.113
Age (years)					
18–49	2046	Reference			
50–59	1467	0.047	1.049	0.849–1.295	0.659
BMI					
Underweight	194	Reference			
Normal	1957	0.238	1.269	0.847–1.901	0.247
Overweight	1078	0.297	1.346	0.878–2.062	0.173
Obese	283	0.397	1.487	0.871–2.537	0.146

### Correlation analysis between daily iodine and salt intake

We also found a positive correlation between iodine intake and salt intake in pooled adults and women of childbearing age (*r* = 0.423, *P* < 0.01; *r* = 0.406, *P* < 0.01). Table 5 shows the correlation coefficients between iodine and salt intake in each area, including the coastal rural area (*r* = 0.369, *P* < 0.01; *r* = 0.350, *P* < 0.01).

**Table 5:** Correlation coefficients of daily iodine and salt intake in different geographical regions.

Region	Women of childbearing age			Pooled		
	N	*r*	r*	N	*r*	r*
Coastal urban area	399	0.409^##^	0.077^##^	1185	0.402^##^	0.092^##^
Coastal rural area	290	0.350^##^	0.164^##^	1002	0.369^##^	0.163^##^
Non-coastal urban area	237	0.434^##^	0.189^##^	746	0.496^##^	0.949^##^
Non-coastal rural area	156	0.471^##^	0.551^##^	580	0.464^##^	0.268^##^
Pooled	1082	0.406^##^	0.102^##^	3513	0.423^##^	0.619^##^

*r* correlation coefficient.

*Adjusted for age, BMI, and (or) gender factors by partial correlation.

^##^Significantly positive correlations between daily iodine and salt intake in each area as detected by Spearman rank correlation analysis (*P* < 0.01).

After adjusting for age, BMI, and (or) gender factors, the correlation coefficients remained statistically significant in each area (all *P* < 0.01).

### Focus on iodine and salt intake among women of childbearing age

**Figure 1:**
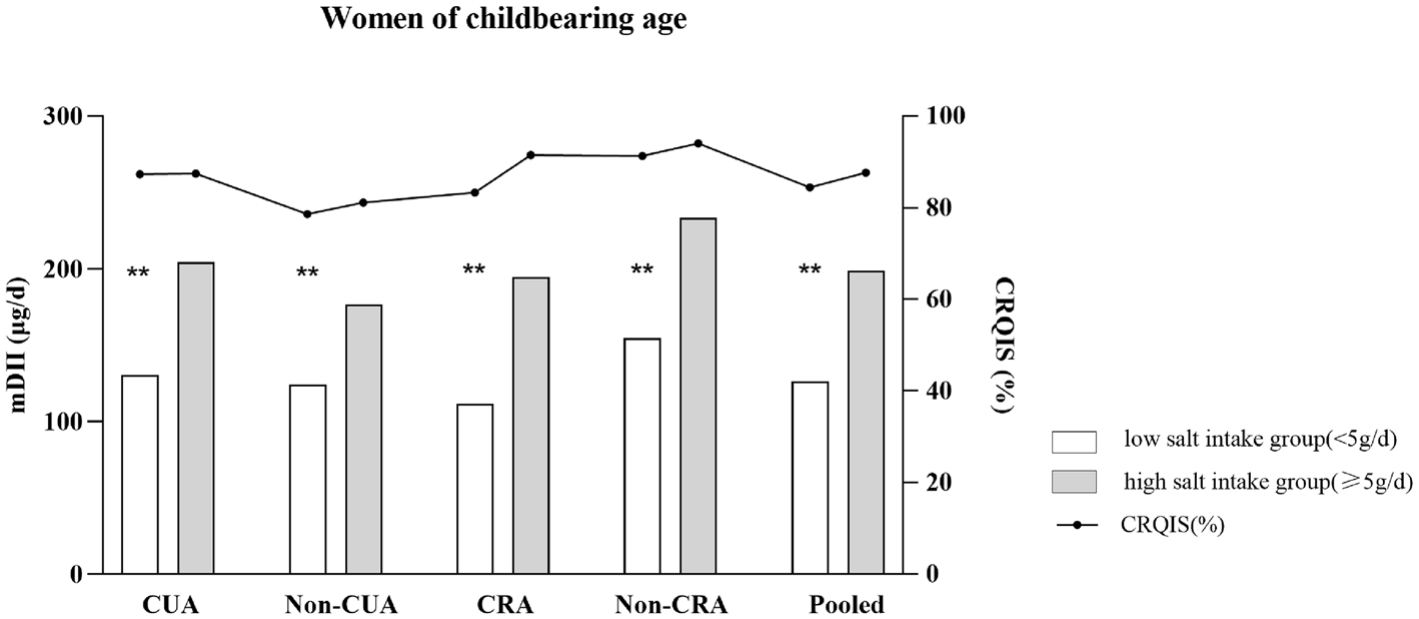
mDII: median daily iodine intake; CRQIS: The consumption rate of qualified iodized salt; CUA: costal urban area; Non-CUA: non-costal urban area; CRA: costal urban area; Non-CRA: non costal rural area: **DII of women of childbearing age with low salt intake group (< 5 g/d) was significantly lower compared to those with high salt intake group (≥ 5g/d) in each area (*P* < 0.001) by Wilcoxon test.

The mDII among women of childbearing age in the low–salt intake group (< 5 g/d) in coastal urban area, coastal rural area, non-coastal urban area, non-coastal rural area, and pooled adults was 130.3 (82.9–159.2), 124.3 (83.5–180.0), 111.7 (62.4–205.7), 154.7 (108.7–239.7), and 126.1 (83.8–183.6) µg/d, respectively. The DII was lower among women of childbearing age in the low–salt intake group (< 5 g/d) compared with the high–salt intake group (≥ 5 g/d) (*P* < 0.05). The coverage of qualified iodized salt among those in the low–salt intake group (< 5 g/d) was 87.3%, 78.6%, 83.3%, 91.3%, and 84.4% in the aforementioned areas, respectively (Fig. 1).

## Discussion

This study showed that the lowest mUIC in Fujian adults aged 18–59 years decreased from 199.9 μg/L in 2009^7^ to 166.1 μg/L in 2017^7^ and 132.0 μg/L in 2021–2022. However, these results indicated that the iodine level in Fujian adults was still adequate. The DII is a more accurate indicator of individual iodine nutrition than UIC^28^, and the mDII in Fujian adults was 195.7 μg/d. We also found that the UIC and DII levels varied across different geographical regions. In particular, noncoastal areas (especially non-coastal rural areas) had the highest iodine intakes compared with coastal areas. The non-coastal rural areas had the highest mUIC in adults compared with the other three areas, and these results were in general agreement with mUICs reported in 2017^7^. The surveys in other countries demonstrated that mUICs were the highest in the coastal urban areas compared with the non-coastal rural area because of higher consumption of aquatic foods. However, foods of sea origin contributed minimally to iodine intake in the coastal areas of China^29^. It was possible that people in coastal areas mistakenly believed that they had taken enough iodine from seafood and no need to consume iodized salt. Another explanation is that people in coastal areas tend to have lighter tastes and lower salt intake^20^.

In this study, the mUIC (134.5 µg/L) and mDII (192.3 µg/d) in nonpregnant, nonlactating women aged 18–49 years reflected adequate iodine intake. Maternal iodine status is particularly important during the first trimester of pregnancy because the embryo relies solely on maternal iodine and thyroid function for normal development. Thyroid hormone deficiency in the first trimester of gestation has been found to have detrimental effects on neuronal migration, cortical lamination and callosal projections in rodent development and on visual attention and visual processing in humans^30^. According to recent studies, pregnant women who regularly consume adequate iodine before they become pregnant have better thyroid profiles than those who began iodine supplementation after pregnancy^9,31^. Hence, it is important for women of childbearing age to consume an adequate amount of iodine, corresponding to the recommended iodine intake of 154.7 μg/d^32^. The iodine level varies globally in women of childbearing age. For instance, Filipino and Australian women were found to have sufficient iodine intake based on the mUIC values of 123.0 and 117 µg/L, respectively^33,34^. In contrast, mild iodine deficiency (ID) was observed among women of childbearing age in Sweden^35^ and the United States^36^. The mUIC and mDII values in women of childbearing age who lived in non-coastal rural area were higher than those in the other three areas, consistent with the overall population. Although a few studies reported better iodine nutrition in coastal areas^37,38^, no specific geographic foci could be assumed to be indicative of low-prevalence areas. Living on the seacoast did not guarantee iodine sufficiency. In fact, other studies revealed that proximity to the coast had no impact on iodine levels^39,40^.

The results showed that the mDSI (10.2 g/d) in Fujian residents aged 18–59 years was two times than the salt intake recommended by the WHO (5 g/d). From an individual perspective, 86.4% of Fujian residents had a salt intake higher than the 5 g/d level. The mDSI in adults aged 18–59 years in this study decreased by 46% compared with 17.6 g/d, which was reported in the CHNS from 2009 to 2011^14^. This finding might be associated with the salt control program^15^, increased publicity of the program in mainstream media, and free distribution of salt control scoops. All these factors encouraged Fujian residents to consciously focus on their daily salt consumption, guiding the development of improved health behaviors. However, although the total salt intake in Fujian adults is far below the national average, unfortunately, it far exceeds the WHO-recommended standard, which states that the adult’s DSI should be less than 5 g. The Chinese dietary reference intake standard states that the recommended nutrient intake of salt should be 5 g/d in adults because it is likely that noncommunicable chronic disease can be prevented if the intake does not exceed 5 g/d^17^. A British study showed that a high-salt diet led to obesity, independent of energy or sugar intake^41^. The risk of childhood and adult obesity increased by 28 and 26%, respectively, with an increased salt intake of 1 g/d^41^. Furthermore, a meta-analysis of 18 cross-sectional studies showed that the higher a person’s sodium intake, the greater their BMI and waist circumference^42^. However, the results of this study showed no increase in the BMI of Fujian residents with DSI by multivariate logistic regression analyses, perhaps because participants aged 18–49 years have more health awareness and prefer to have healthier dietary patterns such as low salt diet than older adults^43^. In this study, the DII was found to be positively correlated with the DSI among the overall population and women of childbearing age in different geographical regions (especially noncoastal areas), which was similar to the findings in Switzerland^25^ and Belgium^44^. Compared with the noncoastal rural areas (152.5 µg/d), the mDII was less than 154.7 µg/d among women of childbearing age with DSI < 5 g/d in the other three areas, which was associated with the consumption rate of qualified iodized salt (< 90%). In China, sodium comes mainly from the salt added during cooking (63.6%)^13^. The comparable mUIC values in this study and those reported in 2017^7^ indicated that the salt reduction strategy had a slight effect on overall iodine intake of Fujian adults. Therefore, public health initiatives are needed to reduce the amount of cooking salt and continue consuming qualified iodized salt in Chinese adults, especially women of childbearing age.

The strength of this study was that the results were based on a large, representative sample of adults in Fujian with measured weight and height data. Their daily iodine and sodium intakes were estimated through reliable measurements of iodine and sodium levels in vivo rather than through dietary recall questionnaires and condiment weighing surveys in vitro. The limitations of the study included the use of spot urine samples, especially for determining salt intake, the lack of data for urine volume in 24 h and urinary potassium excretion, and the lack of reliable data on salt intake for school-age children, pregnant women, and lactating women.

## Conclusions

The findings of this study suggest that the iodine nutrition in the adult aged 18–59 years, nonpregnant population was still adequate after implementing the salt reduction program. The salt intake was generally substantially higher than the WHO recommendations. Further policy implementation is needed to reduce salt intake and continue improvement in monitoring iodine level in Fujian adults in China.

## Data Availability

Details of how to access the data are available from the first author and the correspondence author.

## Abbreviations

IDD: Iodine deficiency disorders
mUIC: Median urine iodine concentration
UIC: Urine iodine concentration
DII: Daily iodine intake
UNaC: Urinary sodium concentration
DSI: Daily salt intake
CDC: Centre for Disease Control
WHO: World Health Organization
IQR: Interquartile range
